# Alteration of mitochondrial homeostasis is an early event in a *C. elegans* model of human tauopathy

**DOI:** 10.18632/aging.203683

**Published:** 2021-11-09

**Authors:** Konstantinos Palikaras, Kavya Achanta, Seoyun Choi, Mansour Akbari, Vilhelm A. Bohr

**Affiliations:** 1 Department of Cellular and Molecular Medicine, Center for Healthy Aging, University of Copenhagen, Copenhagen, Denmark; 2 Department of Physiology, Medical School, National and Kapodistrian University of Athens, Athens, Greece; 3 DNA Repair Section, National Institute on Aging, National Institutes of Health, Baltimore, MD 21224, USA

**Keywords:** aging, Alzheimer’s disease, *C. elegans*, energy metabolism, mitochondria, tau, tauopathy

## Abstract

Tauopathies are a group of progressive neurodegenerative disorders characterized by the presence of insoluble intracellular tau filaments in the brain. Evidence suggests that there is a tight connection between mitochondrial dysfunction and tauopathies, including Alzheimer’s disease. However, whether mitochondrial dysfunction occurs prior to the detection of tau aggregates in tauopathies remains elusive. Here, we utilized transgenic nematodes expressing the full length of wild type tau in neuronal cells and monitored mitochondrial morphology alterations over time. Although tau-expressing nematodes did not accumulate detectable levels of tau aggregates during larval stages, they displayed increased mitochondrial damage and locomotion defects compared to the control worms. Chelating calcium restored mitochondrial activity and improved motility in the tau-expressing larvae suggesting a link between mitochondrial damage, calcium homeostasis and neuronal impairment in these animals. Our findings suggest that defective mitochondrial function is an early pathogenic event of tauopathies, taking place before tau aggregation and undermining neuronal homeostasis and organismal fitness. Understanding the molecular mechanisms causing mitochondrial dysfunction early in tauopathy will be of significant clinical and therapeutic value and merits further investigation.

## INTRODUCTION

Tau is a microtubule-associated protein (MAP) that plays an important role in the assembly and stabilization of microtubules. It is predominantly an axonal protein and is involved in the regulation of neuronal morphology, neurite extension and axonal transport of organelles [1–4]. The pathological aggregation of the microtubule associated tau protein into filaments is a histopathological characteristic of tauopathies, including Alzheimer’s disease (AD) [5, 6]. However, recent studies suggest that soluble tau oligomers, the precursors to the higher order paired helical filaments (PHFs) and neurofibrillary tangles (NFTs), are more toxic and have a greater potential in spreading the tau pathology [7–9]. Moreover, it is also documented that the levels of tau oligomerization are elevated prior to NFTs formation in the brain of AD patients supporting a dynamic relationship between tau oligomerization and the progression of tauopathy in AD brains [10, 11].

Aging is one of the greatest risk factors for the development and progression of neurodegenerative diseases, including tauopathies [12, 13]. Moreover, multiple studies have shown an interconnection between tau pathology and mitochondrial dysfunction [14, 15]. Indeed, tau-mediated mitochondrial damage could be due to reduced levels of mitochondrial activity [16–18], its interaction with mitochondrial proteins [11], modulation of mitochondrial dynamics [19] and activation of the mitochondrial apoptotic pathway [20–22]. Mitochondria constantly undergo fusion and fission to regulate their size, shape and numbers in response to nutrient availability, stress conditions and energy demand of the cell [23]. Neurons are particularly vulnerable to impairment of mitochondrial dynamics because they are largely dependent on mitochondria for energy production, especially at the synapses and because distribution of mitochondria to distal parts of the neuron require energy [24–27]. Mitochondrial fusion and fission events are also quality control mechanisms, since fusion complements damaged components of a mitochondrion with those of a healthy mitochondrion, and mitochondrial fission facilitates the selective autophagic removal of defective organelles, known as mitophagy [28]. Previous studies have shown that tau accumulation and mislocalization disturbs microtubule stability and interferes with transport of neuronal organelles, including mitochondria. These disturbances in trafficking partly explain tau mediated synapse and memory deficits in tauopathies [6, 8]. Although the effect of tau lesion on mitochondrial metabolism is frequently reported, it remains unclear whether altered mitochondrial homeostasis is a result of fully developed tau pathology or it is an early event in tauopathies and as such may play a critical role in the disease progression.

Here, we report that excessive mitochondrial damage is an early event in neuronal cells in a nematode model of human tauopathy. Transgenic animals expressing the full length of wild type tau displayed a reduced number of mitochondria in neuronal processes of *C. elegans* larvae. The morphological characteristics of mitochondria indicated that tau expression promotes deregulation of mitochondrial function during nematode development and adulthood. Despite the fact that tau aggregation was mainly observed in adult animals, locomotion deficits were also present during larval stages underscoring the toxic effects of tau in organelle function and cellular physiology. Finally, we found that chelating calcium by ethylene glycol tetraacetic acid (EGTA) increased the mitochondrial membrane potential and improved motility in the tau-expressing larvae suggesting a positive correlation between mitochondrial function, calcium homeostasis and neuronal performance.

## RESULTS

### Age-dependent aggregation of wild type tau in *C. elegans* neurons

**Figure 1:**
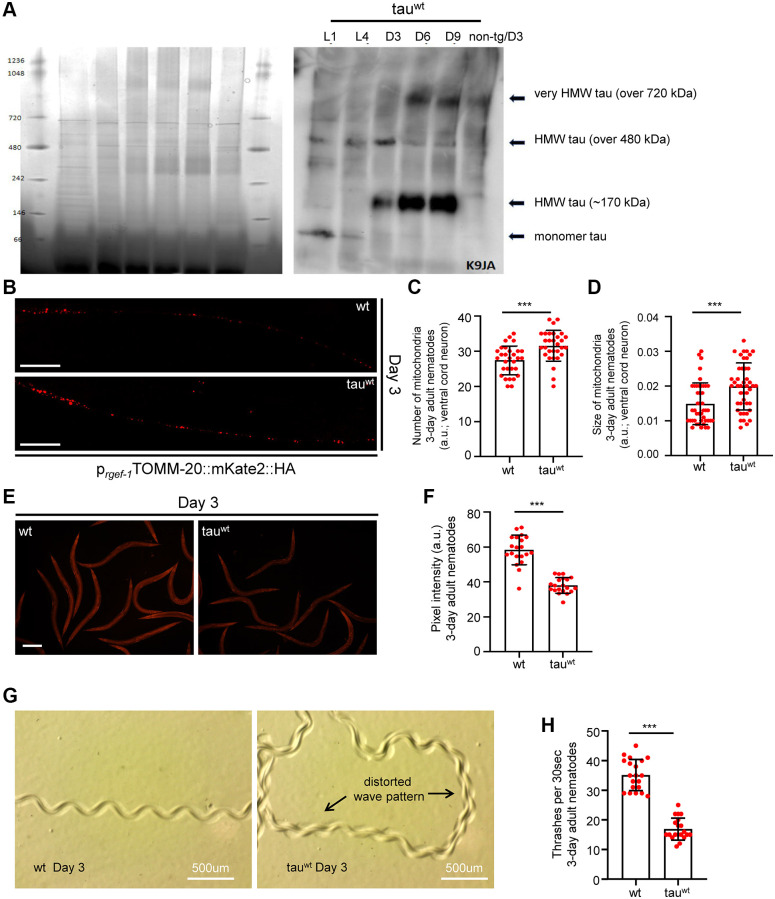
**Impaired mitochondrial homeostasis and motility defects in tau^wt^-expressing 3-day adult animals.** (**A**) Age-dependent accumulation of wild type tau protein in neuronal cells (heigh molecular weight; HMW). (**B**) Representative fluorescent images of transgenic nematodes expressing pan-neuronally mitochondria-targeted mKate2::HA. Scale bar, 20 μm. Tau^wt^-expressing 3-day-adult nematodes display (**C**) increased mitochondrial population and (**D**) smaller organelle compared to their wild type counterparts (*n* = 30; ^***^P < 0.0001; unpaired *t*-test). (**E**, **F**) Tau^wt^ expressing nematodes display decreased mitochondrial membrane potential (*n* = 40; ^***^P < 0.0001; unpaired *t*-test). Scale Bar, 100 μm. (**G**) Representative images of 3-day adult wild type and tau^wt^ expressing nematodes tracks. (**H**) Body bends of 3-day adult wild type and tau^wt-^ animals per 30 seconds in M9 buffer (*n* = 20; ^***^P < 0.0001; unpaired *t*-test).

To investigate the impact of tau on mitochondrial activity, neuronal function and organismal physiology, we utilized an already characterized nematode strain that expresses the full length of wild type human tau protein under the control of the pan-neuronal promoter of the *snb-1* gene (the nematode synaptobrevin homologue) [29]. To avoid possible toxic effects of tau overexpression, we used a transgenic nematode strain expressing wild type tau at low levels (tau^wt-low^; PIR3, henceforth tau-expressing nematodes), which display mild phenotypic abnormalities [29]. We collected age-synchronized worms and performed total tau protein extraction from the transgenic nematodes to assess the formation of tau oligomers and aggregates over time. We found that high molecular weight tau oligomers started to accumulate from day three of adulthood and gradually increased in *C. elegans* neurons with age (Figure 1A and Supplementary Figure 1A–1B). Accumulation of tau protein with age was not due to altered transcriptional activity of the tissue specific *snb-1* promoter (Gene Expression Omnibus (GEO) dataset GSE832 [30]).

A recent study demonstrated that a low level of tau expression impaired mitochondrial number and distribution in neuronal processes in the tau-expressing *C. elegans* strain during day 1 and day 3 of adulthood [29]. To further examine the effect of low level of tau expression on neuronal mitochondria integrity, we generated transgenic animals co-expressing mitochondria-targeted mKate2::HA with wild type tau in neuronal cells. Interestingly, we found that 3-day-old tau-expressing animals displayed more and smaller axonal mitochondria compared to the age-matched wild type nematodes (Figure 1B–1D and Supplementary Figure 2A). A growing body of evidence suggests that challenging conditions trigger mitochondrial fragmentation generating smaller organelles to promote the isolation and subsequent removal of damaged mitochondria through mitophagy [31–34]. Notably, several mutant isoforms of tau have been shown to inhibit mitophagy leading to the accumulation of defective mitochondria and subsequently to cellular and tissue deterioration [35, 36]. Thus, our finding suggests that fragmented and damaged mitochondria accumulate in the neurons of tau-expressing worms. Although mitochondrial activity gradually declined with age in wild type animals, the mitochondrial membrane potential was highly reduced even in young tau-expressing nematodes (Figure 1E–1F and Supplementary Figure 2B), underlining the toxic effect of tau expression on energy metabolism.

Proprioception has an essential role in the movement coordination and body balance of an organism. Age-dependent deterioration of biological systems and pathological conditions could cause proprioception impairment leading to uncontrolled and inefficient mobility [37]. In *C. elegans*, proprioception can be evaluated by monitoring the sinusoidal wave pattern that is generated by the periodic bending of its head and body [38, 39]. Wild type nematodes inscribe a sinusoidal track as they move on an agar plate seeded with OP50 *E. coli* bacterial strain. The characteristic properties (amplitude and wavelength) of tracks generated by the tau-expression animals were dramatically perturbed indicating severe motility deficits (Figure 1G). Moreover, we assessed the locomotion by measuring bending behavior in both wild type and the tau-expressing nematodes during adulthood. Different age groups of wild type and transgenic nematodes were placed in a 10 μl droplet of M9 buffer and were allowed to swim freely for 1 minute, to become accustomed to their new environment. Body bends were then monitored for 30 seconds. In accord with the abnormal sinusoidal tracks, the tau-expressing nematodes displayed pronounced locomotion defects throughout adulthood compared to the wild type animals (Figure 1H and Supplementary Figure 2C). These results suggest that mitochondrial dysfunction in the tau-expressing nematodes could mediate energy deprivation subsequently leading to neuronal deregulation and eventually to motility defects.

### Impaired activity of neuronal mitochondria and motility defects in larval stages

**Figure 2:**
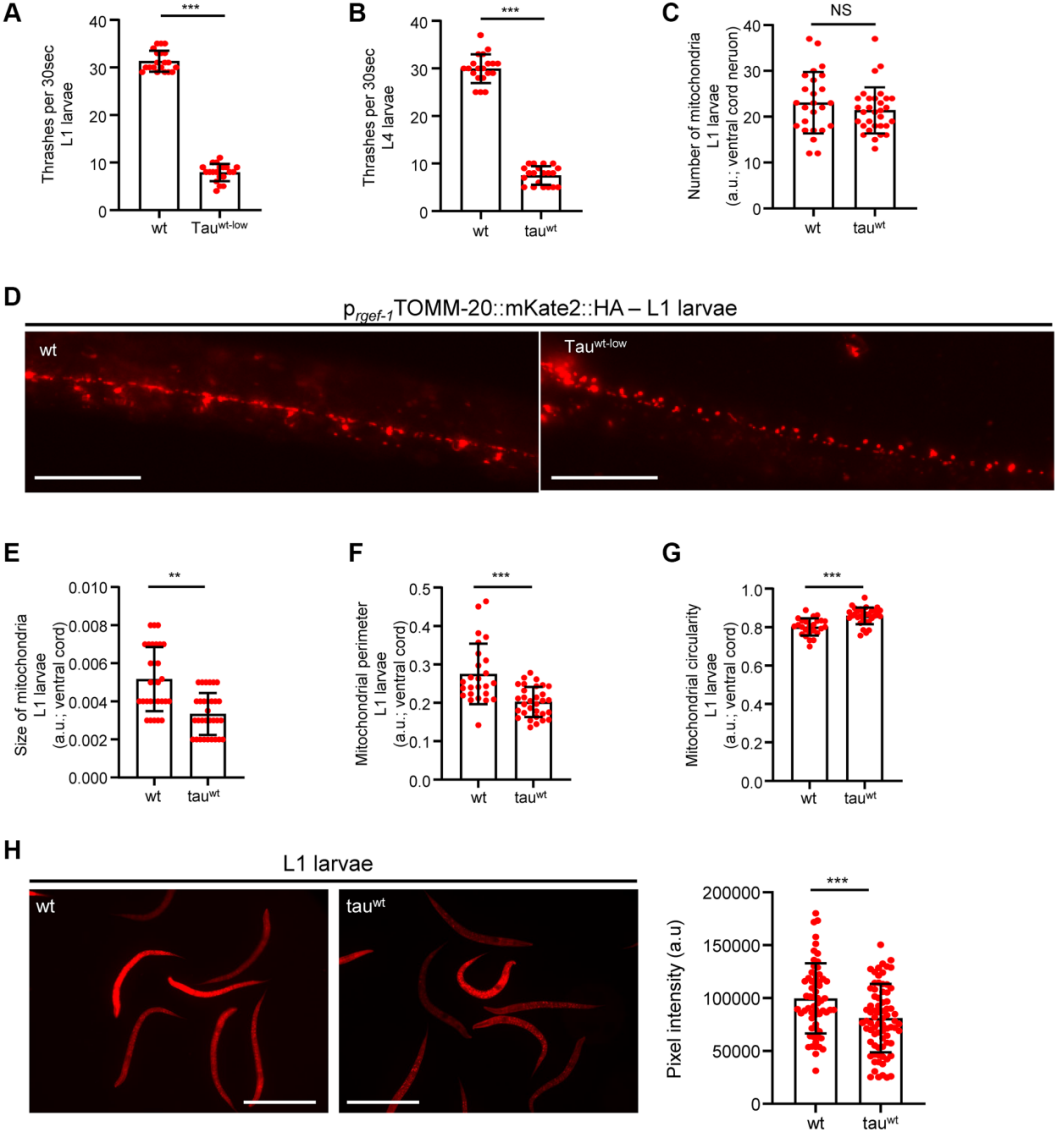
**Altered mitochondrial morphology and activity in tau^wt^-expressing larvae.** Wild type and tau^wt^ L1 (**A**) and L4 (**B**) larvae body bends per 30 seconds in M9 buffer. (**C**) Mitochondrial population in the ventral nerve cord of L1 nematodes. (**D**) Representative fluorescent images of transgenic nematodes expressing pan-neuronally mitochondria-targeted mKate2::HA. Scale bar, 20 μm. Tau^wt^-expressing L1 larvae display (**E**) smaller, (**F**, **G**) more circular and (**H**) Fewer active mitochondria compared to wild type animals. Scale bar, 100 μm. (*n* = 50; NS *P* > 0.05, ^**^P < 0.001, ^***^P < 0.0001; unpaired *t*-test).

**Figure 3:**
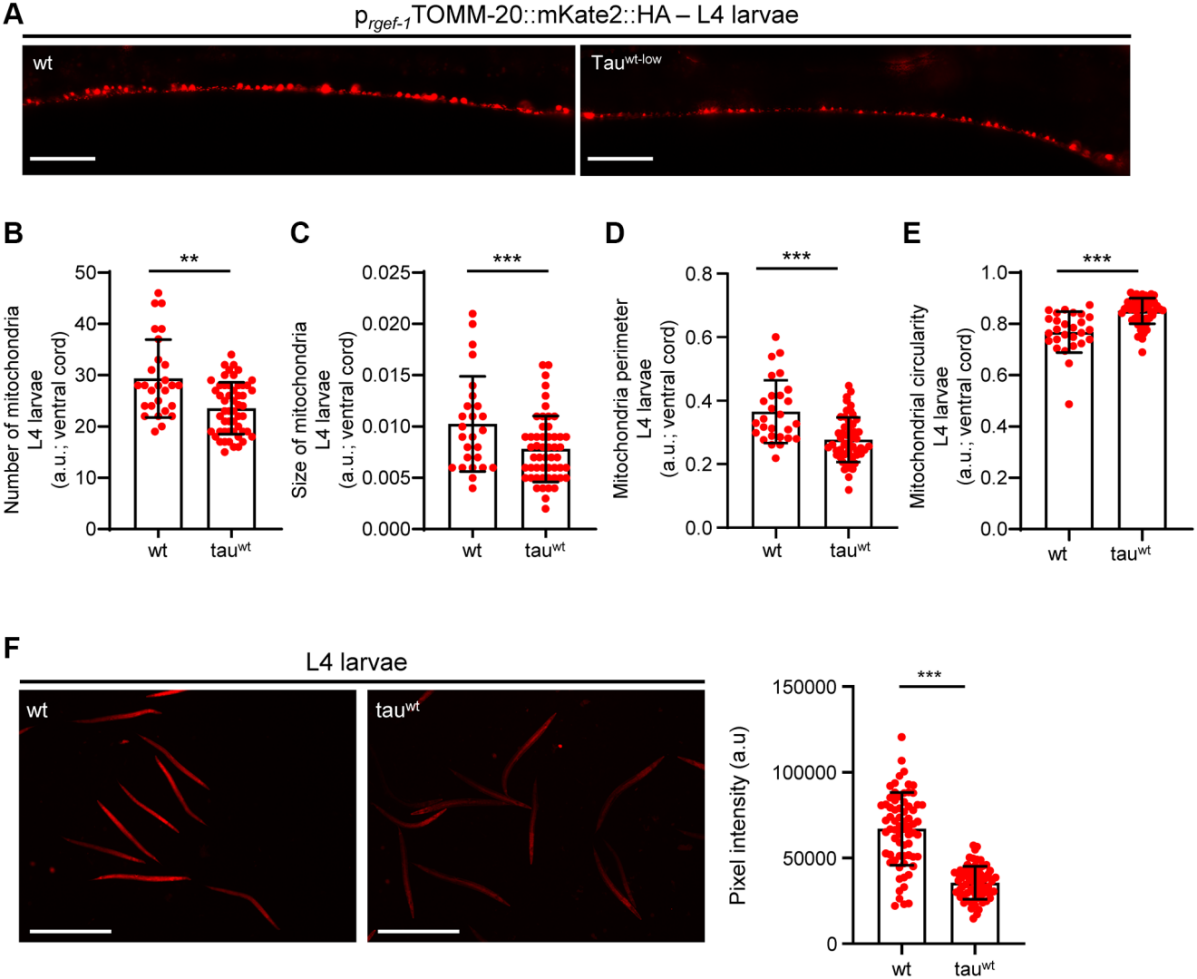
**Tau^wt^-expressing L4 nematodes display impaired mitochondrial morphology and function.** (**A**) Representative fluorescent images of L4 transgenic nematodes expressing panneuronally mitochondria-targeted mKate2::HA. Scale Bar, 20 μm. (**B**) Mitochondrial population in the ventral nerve cord of L4 nematodes. Neuronal processes of tau^wt^-expressing L4 larvae display (**C**) smaller, (**D**, **E**) more circular and (**F**) fewer active mitochondria compared to wild type animals (*n* = 30–50; ^**^P < 0.001, ^***^P < 0.0001; unpaired *t*-test).

Accumulating evidence underscores the effect of critically high levels of tau and tau lesions on mitochondrial homeostasis [40, 41]. Tau may influence mitochondrial function both directly via its localization on mitochondrial compartments and indirectly through perturbation of cytoskeleton components [40–47]. While the association between tau levels and energy metabolism is established, it is not clear whether mitochondrial dysfunction is an early pathological feature of high levels of tau or a consequence of its excessive formation of protein aggregates. To discriminate between these two scenarios, we focused on the L1 and L4 larval developmental stages. Although the formation of tau oligomers and/or aggregates was not detectable in neuronal cells of L1 and L4 larvae (Figure 1A), tau-expressing animals displayed a reduction in body bends compared to the wild type nematodes (Figure 2A–2B). We next monitored the mitochondrial population in the neuronal processes of the ventral and dorsal cord of both the L1 and L4 larvae, which expressed pan-neuronally mitochondria-targeted mKate2::HA and wild type tau. We found decreased mitochondrial density in ventral (Figure 2C–2D; Figure 3A, 3B) and dorsal cord neurons in larval stages (Supplementary Figure 3A–3B, 3F–3G).

To investigate whether the locomotion deficits were generated because of excessive mitochondrial damage, we evaluated several aspects of mitochondrial morphology, including size, perimeter and circularity, which have been used to evaluate mitochondrial dysfunction [48–51]. Tau-expressing larvae presented smaller and more globular mitochondria compared to the wild type counterparts (Figure 2E–2G, Figure 3C–3E and Supplementary Figure 3C–3E, 3H). Moreover, mitochondrial membrane potential assessment of the tau-expressing nematodes displayed fewer active organelles indicating that tau expression is sufficient to alter not only the shape of mitochondrial networks but also their activity during early development (Figure 2H and Figure 3F). These results indicate that increased mitochondrial damage and impaired energy generation might drive neuronal dysfunction and the abnormal locomotion of the tau-expressing nematodes and highlight mitochondrial defects as an early pathological feature of tauopathy.

### Calcium homeostasis modulates mitochondrial activity and neuronal fitness in tau-expressing nematodes

Ca^2+^ is an important second messenger that controls multiple cellular processes. Local Ca^2+^ signals are widely recognized as broad regulators of neuronal function and survival [52, 53]. Neurons are highly dependent on balanced Ca^2+^ homeostasis, since they have developed intricate regulatory mechanisms coupling Ca^2+^ signaling with their molecular and biochemical machineries. Indeed, altered cytosolic Ca^2+^ fluctuations lead to impaired neurotransmission, axon guidance, spine formation and subsequently to neuronal loss and cognitive dysfunction [54, 55].

**Figure 4:**
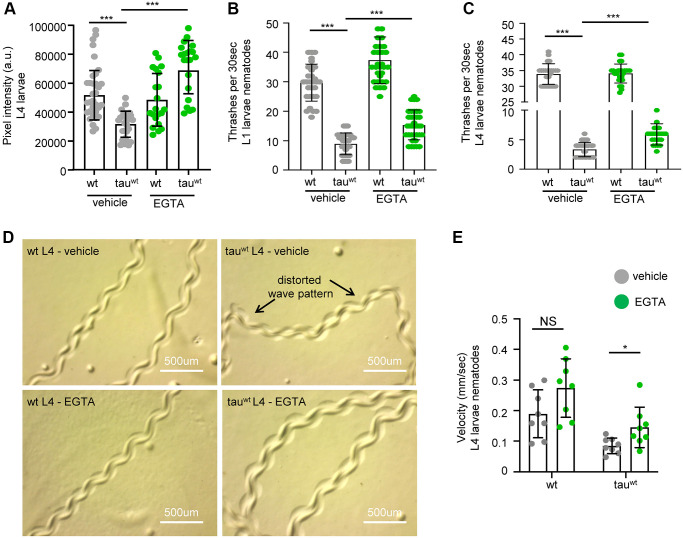
**Cytoplasmic calcium chelation rescues motility defects in tau^wt^-expressing larvae.** (**A**) Mitochondrial activity is increased in tau^wt^-expressing L4 nematodes upon 10 mM EGTA supplementation. Locomotion defects are ameliorated in both (**B**) L1 and (**C**) L4 tau^wt^-expressing nematodes in response to 10 mM EGTA treatment (*n* = 50; ^***^P < 0.0001; unpaired *t*-test). (**D**) Representative images of wild type and tau^wt^-expressing nematodes tracks with or without 10 mM EGTA treatment. (**E**) Velocity assessment of wild type and tau^wt^-expressing nematodes with or without 10 mM EGTA treatment (*n* = 8; NS *P* > 0.05, ^*^P < 0.03; unpaired *t*-test).

A recent study in *C. elegans* demonstrated that overexpression of a mutant isoform of tau (tau^A152T^) promoted necrotic cell death of glutamatergic neurons through dysregulation of the cytosolic calcium levels [56]. Therefore, we examined whether calcium deregulation is implicated in the impairment of mitochondrial activity in the tau-expressing nematodes. Interestingly, supplementation with 10 mM EGTA, a calcium chelating agent, restored mitochondrial membrane potential in tau-expressing animals without any detectable effect on wild type worms (Figure 4A). Moreover, the bending behavior, the sinusoidal wave pattern and the velocity of tau-expressing L4 larvae were improved following calcium chelation without any substantial effect on the behavior in wild type (Figure 4B–4E and Supplementary Figure 4A). However, EGTA treatment could not rescue the severe motility defect in the tau-expressing nematodes during adulthood (Supplementary Figure 4B). Moreover, EGTA supplementation did not affect tau oligomer and aggregate formation, suggesting that calcium chelation solely impacts mitochondrial homeostasis (Supplementary Figure 5A–5B). These findings suggest that tau expression results in altered calcium homeostasis enhancing neuronal vulnerability to degeneration during aging, and demonstrate tight interplay between tau overexpression, calcium homeostasis, and mitochondrial function, in neuronal health.

## DISCUSSION

Aging is universally associated with a marked decrease in brain function and increased susceptibility to neurodegeneration. In human populations, this is manifested as an ever-increasing prevalence of devastating neurodegenerative conditions, including AD and other types of dementia. Therefore, the development of novel therapeutic interventions against human aging and age-related pathologies is a top research priority.

Mitochondrial dysfunction is a recognized hallmark of aging and age-associated diseases [12, 57]. Recent studies in AD mouse models suggest that Aβ plaques and tau tangles formation are accompanied with mitochondrial dysfunction [58–60]. However, it remains elusive whether impaired mitochondrial metabolism is an early pathological feature of disease development. It is shown that Aβ and tau are localized to mitochondria, where they perturb energy metabolism through either their interference with mitochondrial import machinery or inhibition of mitochondrial enzymes and electron transport chain function [61–63]. Moreover, tau aggregates promote mitochondrial dysfunction and exacerbate Aβ-related mitochondrial damage [18, 64]. Although these results implicate Aβ and tau lesions in energy homeostasis, the role of mitochondrial impairment on the initiation and progression of AD is not well understood.

Our model recapitulates several key features of the human neurodegenerative disorders, including adult onset, progressive neurodegeneration, accumulation of abnormal tau, and shorter lifespan. The cumulated results of our study indicate that, at least in this tauopathy model, neurotoxicity depends on protein alterations and mitochondrial dysfunction, which takes place before the formation of detectable levels of aggregates during larval stages, underscoring the detrimental neurotoxic effect of high level of tau (Supplementary Figure 6). Tau-expressing nematodes presented an increased number of defective mitochondria in neuronal processes, which may be a result of impaired removal of damaged organelles. Indeed, recent studies in nematodes and mammalian cells have demonstrated that mutant isoforms of tau block neuronal mitophagy and result in pronounced mitochondrial damage and neuronal loss [35, 36]. In sharp contrast, mitochondrial density was markedly reduced in the L1 and L4 tau-expressing larvae suggesting that mitophagy is still efficient in eliminating dysfunctional organelles at least in the larvae. Thus, aging and tau aggregate formation act synergistically to deregulate mitochondrial activity and quality surveillance mechanisms, leading to a gradual accumulation of defective organelles and subsequently to neurodegeneration.

A growing body of evidence demonstrates that calcium homeostasis collapse is a critical modulator of tau-mediated neurotoxicity. Transgenic nematodes expressing the mutant tau isoform A152T (tau^A152T^) displayed progressive degeneration of glutamatergic neurons through impairment of cellular calcium levels that eventually lead to necrotic cell death induction. Interestingly, depletion of the Ca^2+^ binding chaperones calreticulin (CRT-1) and calnexin (CNX-1) and the Ca^2+^ -dependent phosphatase calcineurin (CNB-1) delayed neuronal cell death in L4 larvae [56]. Due to the subcellular localization of CRT-1 and CNX-1 in endoplasmic reticulum (ER), these findings support that tau^A152T^ expression impairs ER homeostasis resulting in enhanced release of Ca^2+^ into the cytoplasm. A very recent study in rat primary cortical neurons and human iPSC-derived neurons documented that tau mutations inhibit mitochondrial Na^+^/Ca^2+^ exchanger (NCLX) function and deregulate mitochondrial calcium efflux, leading to altered cytosolic Ca^2+^ levels and subsequently to increased vulnerability of neuronal cells to cell death. [65]. Therefore, tau mutations could modulate calcium homeostasis by influencing the main cellular storage sites ER and mitochondria.

Although we cannot exclude possible differences in mechanisms of tau toxicity between *C. elegans* and human disease, the enhanced biological toxicity of mutant tau as reported previously, the degenerative nature of the pathology, and the selective accumulation of abnormal tau in areas of neuronal degeneration, all argue that the mechanisms of tau neurotoxicity are conserved between *C. elegans* and humans. Collectively, our findings underline the essential impact of early tau oligomer formation on mitochondrial dysfunction and disease development. Investigating the tight interplay between tau oligomers and energy metabolism will enlighten new avenues for therapeutic strategies to slow or halt the progression of dementia-related diseases such as AD. The *C. elegans* tauopathy model can be used as a screening platform to identify novel genes and chemical compounds that protect against early tau-mediated mitochondrial damage and neurotoxicity.

## MATERIALS AND METHODS

### *C. elegans* strains and culture methods

We followed standard procedures for *C. elegans* strain maintenance (Brenner, 1974 Genetics 77). Nematode rearing temperature was kept at 20°C, unless noted otherwise. The following strains were used in this study: N2: wild-type Bristol isolate, PIR3: *pirIs3*[p_*snb-1*_ htau40^WT-low^; p_*myo-*2_GFP]. To monitor mitochondrial morphology in neuronal cells, we used the following transgenic animals: SJZ216: *foxSi44*[p_*rgef-1*_TOMM-20::mKate2::HA]I and *foxSi44*[p_*rgef-1*_TOMM-20::mKate2::HA]I; *pirIs3*[p_*snb-1*_htau40^WT-low^; p_*myo-*2_GFP]. We used Ethylene glycol tetraacetic acid (EGTA) as a chemical to reduce specifically cytosolic calcium. EGTA was administered at a final concentration of 10 mM. EGTA concentration was prepared by dilutions in 150 ml of sterilized water, from a concentrated stock solution (0.5 M), and applied to the top of the agar medium. Plates were then gently swirled to allow the drug to spread to the entire OP50-seeded NGM surface. Identical drug-free water solutions were used for the control plates. Animals were treated with EGTA for two generations.

### Thrashing assay

Wild type and tau^wt^-expressing L1, L4 and 3-day-adult nematodes were transferred in 10 μl M9 buffer using an eyelash pick. Animals were allowed to swim freely for 1 minute to be accustomed to the new environment. Then, body bends were assessed for 30 seconds. For each experiment, at least 50 animals were examined for each strain/condition. Each assay was repeated at least two times. The Prism software package (GraphPad Software) was used for statistical analyses.

### Mitochondrial imaging

TMRE (tetramethylrhodamine, ethyl ester, perchlorate) is a dye that accumulates in intact, respiring mitochondria. Embryos/eggs were placed and grown at 20°C in the presence of 150 nM TMRE. Stained and washed L1, L4 and 3-day-adult nematodes were immobilized with levamisole before mounting for microscopic examination with a Zeiss AxioImager Z2 epifluorescence microscope. Images were acquired under the same exposure. Average pixel intensity values were calculated by sampling images of different animals. The mean and maximum pixel intensity were calculated for each animal in these images using the ImageJ software (http://rsb.info.nih.gov/ij/). For each experiment, at least 30–50 animals were examined for each strain/condition. The assessment of the mitochondrial morphology was performed by using ImageJ software, as it is previously described [66]. For quantitative characterization of mitochondrial morphology fluorescent images were acquired and used for the analysis. Background was subtracted and the resulting images were thresholded at the default setting. The resulting particles were analyzed and the following parameters were collected for each mito-mKate2-labeled particle: area, perimeter and circularity. Mitochondrial number was evaluated by counting the average number of mito-mKate2-labeled puncta per 100 μm of axonal length. Each assay was repeated at least three times. The Prism software package (GraphPad Software) was used for statistical analyses.

### Worm tracking software

To obtain synchronized populations of worm, 5–7 L4 worms were transferred onto NGM plates (with or without EGTA) and allowed to lay eggs for 24 h. After removing the adult worms, each synchronized progeny was cultured to L4 larvae. For worm imaging, tracking, and describing the worm crawling, synchronized L4 larvae were recorded using the Wormlab software (MBF Bioscience) and captured at rates 15 frames per second. Each worm was manually detected, and their locomotion were analyzed for each frame. The movements were exported to Excel files and worm tracks were generated by GraphPad Prism software. The velocity was evaluated by measuring the distance of each worm that moved for 30 seconds and was calculated by utilizing ImageJ based on the worm track graph. Statistical analysis was performed using GraphPad Prism software.

### Western blot analysis

For total tau protein extraction, age-synchronized worms were washed off NGM plates using M9 buffer. To completely remove the bacteria, the washing steps were repeated thrice. The resulting worm pellets were resuspended in 1X protein sample buffer containing (20 mM Hepes, pH 7.9, 25% glycerol, 0.42 M NaCl, 1.5 mM MgCl2, 0.2 mM EDTA, 0.5 mM DTT) and lysed by sonication (3 × 10 s, 10 s break) on ice. After a brief centrifugation at 40,000 g for 15 min, the supernatants were analyzed on 3–12% native PAGE. The entire extraction procedures were carried out on ice and centrifugation steps were performed at 4°C. All buffers contained Complete Protease and Phosphatase Inhibitor cocktail (Sigma-Aldrich). The proteins were transferred to nitrocellulose membranes and immunoblotted. The following antibodies were used: K9JA (1:5,000; no. A0024; Dako), Anti-Tau (T22), oligomeric antibody (1:1000; ABN454; Sigma-Aldrich). Coomassie staining serves as loading control. For SDS-PAGE, 100 synchronized worms of the indicated ages were washed off NGM plates with M9 buffer and the bacteria were removed in the subsequent washing steps. The resulting worms were allowed to settle under gravity to form a pellet and were resuspended in 100 μl of 2X protein sample buffer containing 355 mM 2- mercaptoethanol and boiled at 96°C for 10 min. The supernatant was collected by brief centrifugation. Proteins were separated in Tris-glycine SDS gel and transferred onto PVDF membrane. Each experiment was done in two biological replicates. The following antibodies were used: Anti-Human Tau (#A0024; Dako), Tau monoclonal antibody (TAU-5) (#AHB0042, Invitrogen) Pan actin (ACTN05 (C4) #MA5-11869; Life Technologies), GAPDH (#sc25778; Santa Cruz Biotechnology).

## Supplementary Materials

Supplementary Figures
