# Supplementary material for: Alteration of mitochondrial homeostasis is an early event in a C. elegans model of human tauopathy
Source: Aging (Albany NY). 2021 Nov 9;13(21):23876–94. doi: 10.18632/aging.203683 (PMC8610126; doi:10.18632/aging.203683)
Supplement: Supplementary Figures [file aging-13-203683-s001.pdf]

SUPPLEMENTARY FIGURES

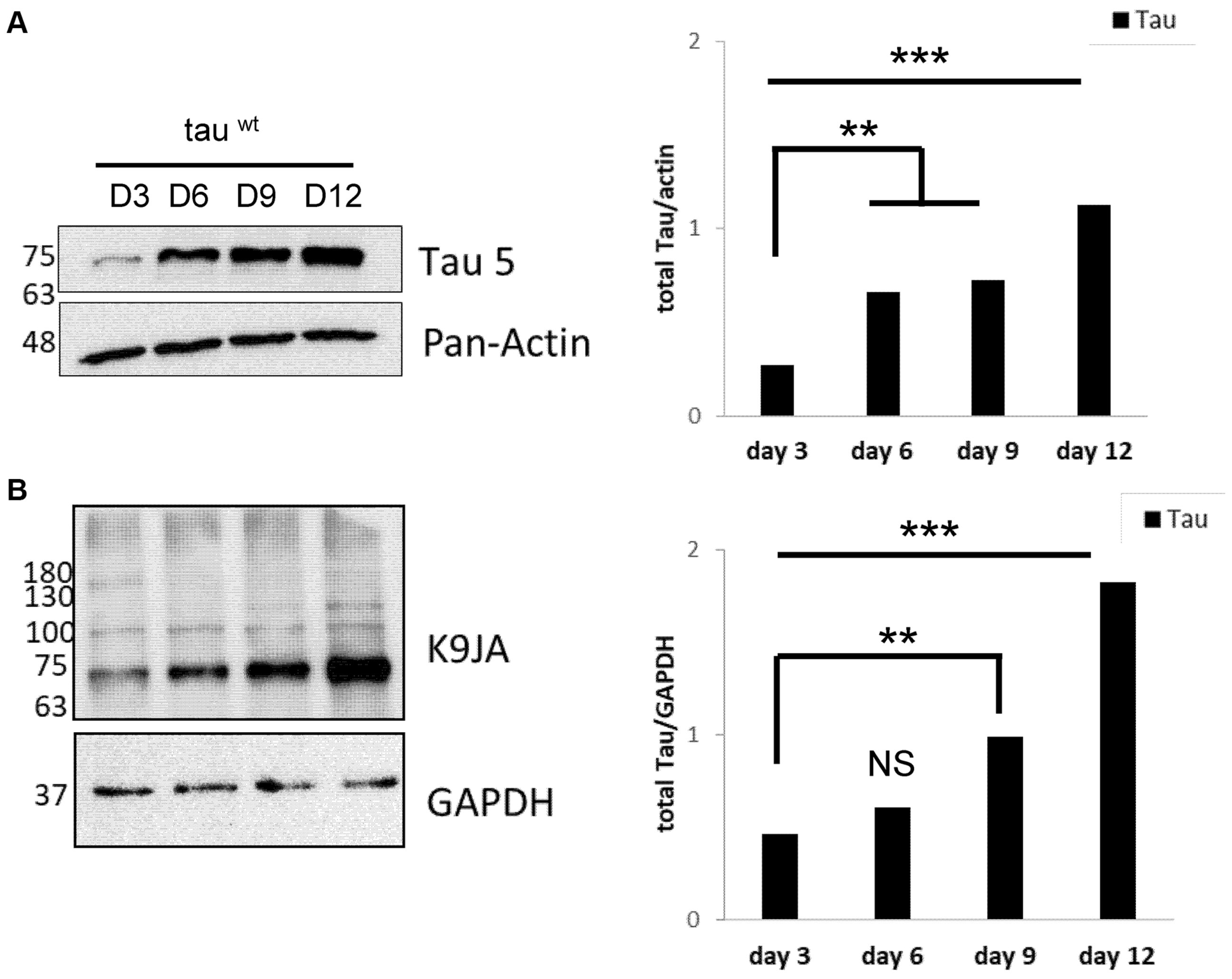

Supplementary Figure 1. The protein levels of tau^{wt} are gradually elevated in neuronal cells with age. (**A**, **B**) Worm extracts prepared from 3-, 6-, 9-day adult transgenic tau^{wt}-expressing nematodes were resolved by SDS-PAGE and immunoblotted with Anti-Tau, Tau 5 and K9JA, antibodies (NS $P > 0.05$, $^{**}P < 0001$, $^{***}P < 0.0001$; one-way ANOVA corrected with Sidak multiple comparison test).

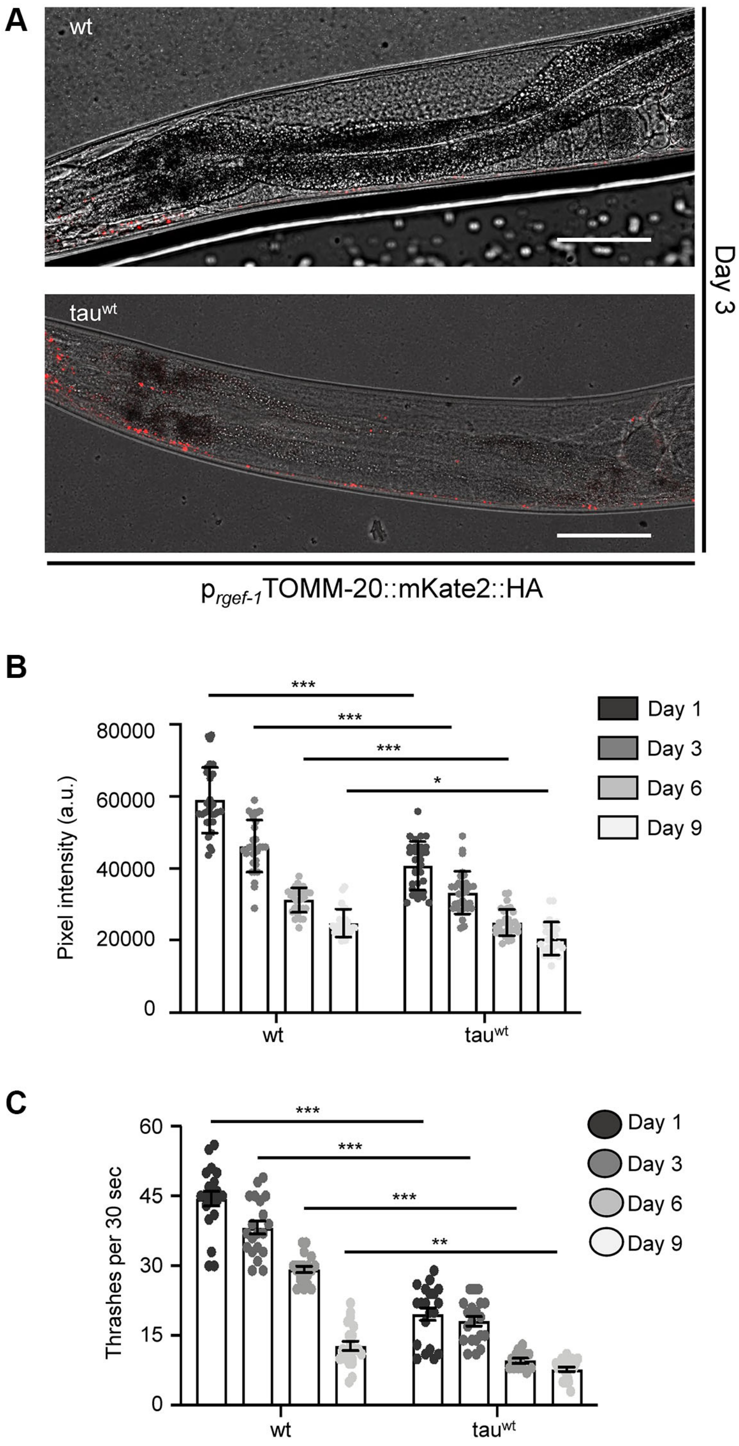

Supplementary Figure 2. (**A**) Representative brightfield and fluorescent images of transgenic nematodes expressing pan-neuronally mitochondria-targeted mKate2::HA. Scale bar, 20 μm (**B**) Mitochondrial membrane potential (TMRE staining) and (**C**) locomotion gradually decline with age in both wild type and tau^{wt}-expressing adult nematodes. (n = 50; NS $P > 0.05$, $^{**}P < 0.0021$, $^{***}P < 0.0002$; two-way ANOVA corrected with Sidak multiple comparison test).

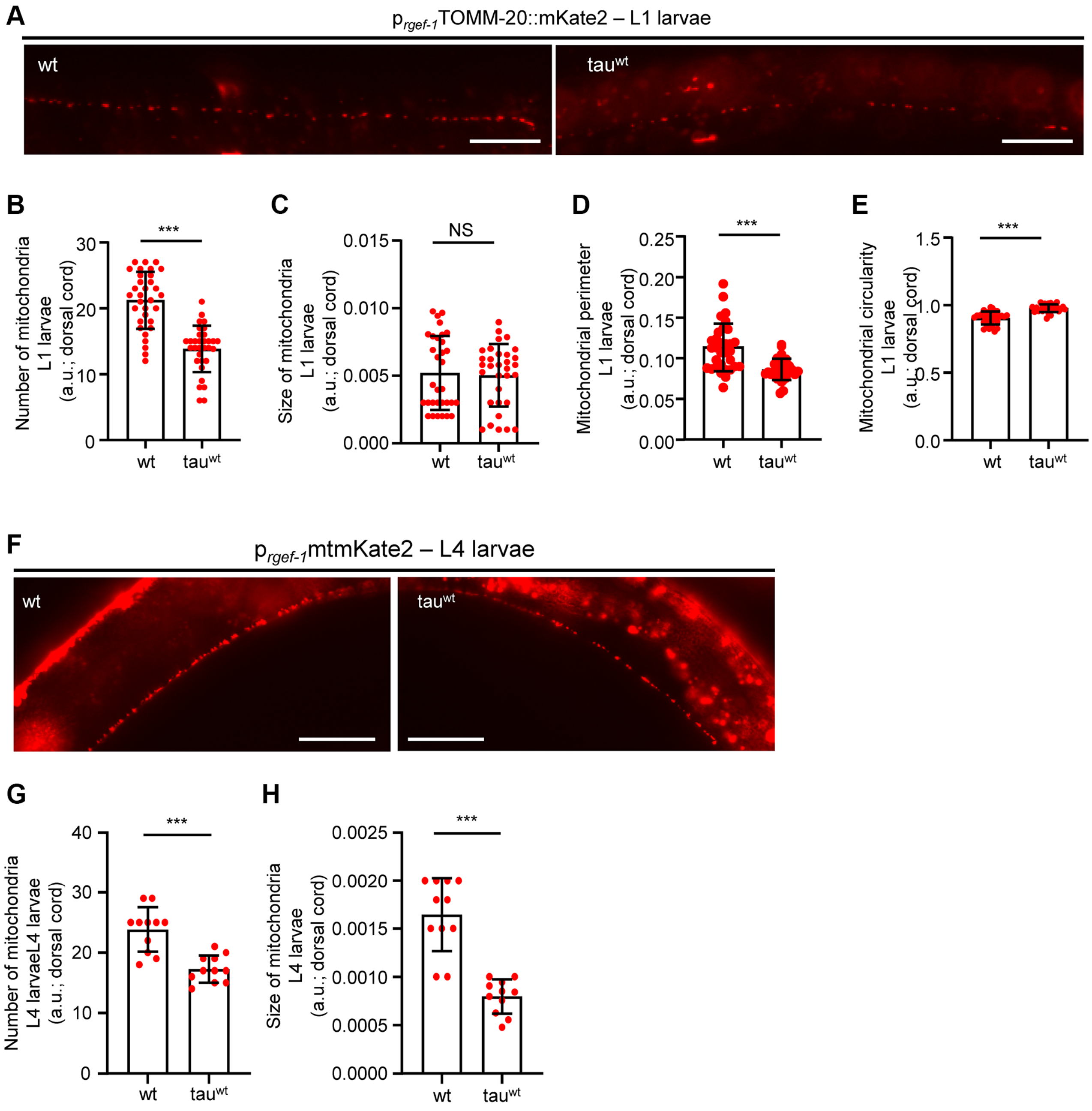

Supplementary Figure 3. Mitochondrial morphology in the dorsal nerve cord of tau^{wt}-expressing larvae. Representative fluorescent images of (**A**) L1 and (**F**) L4 transgenic nematodes expressing pan-neuronally mitochondria-targeted mKate2::HA. Scale Bar, 20 µm. Mitochondrial population in the dorsal nerve cord of (**B**) L1 and (**G**) L4 tau^{wt}-expressing nematodes. Tau^{wt}-expressing larvae display (**C**, **H**) smaller, (**D**, **E**) more circular organelles compared to wild type animals (n = 30–50; NS $P > 0.05$, $^{***}P < 0.0001$; unpaired t-test).

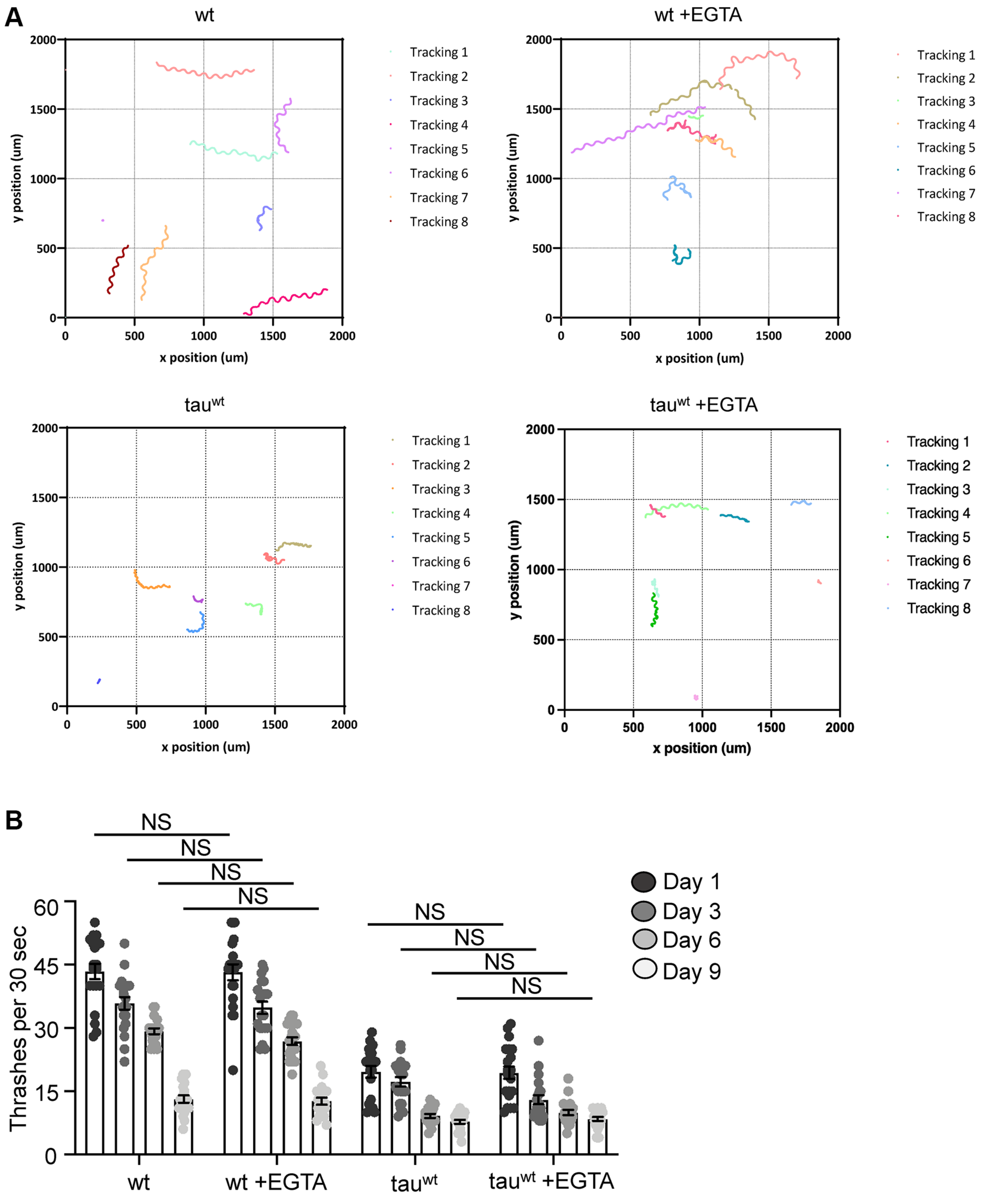

Supplementary Figure 4. Calcium chelation does not restore locomotion defects of tau^{wt}-expressing nematodes during adulthood. (**A**) Tracks of mid-point of wild type and tau^{wt}-expressing nematodes with or without 10 mM EGTA treatment. Animals were allowed to crawl in OP50-seeded NGM plates for 10 minutes. The tracks were generated by using WormLab software. (**B**) Body bends of 1-, 3-, 6- and 9-day wild type and transgenic tau^{wt}-expressing nematodes with or without 10 mM EGTA treatment (n = 20; NS $P > 0.05$, two-way ANOVA corrected with Sidak multiple comparison test).

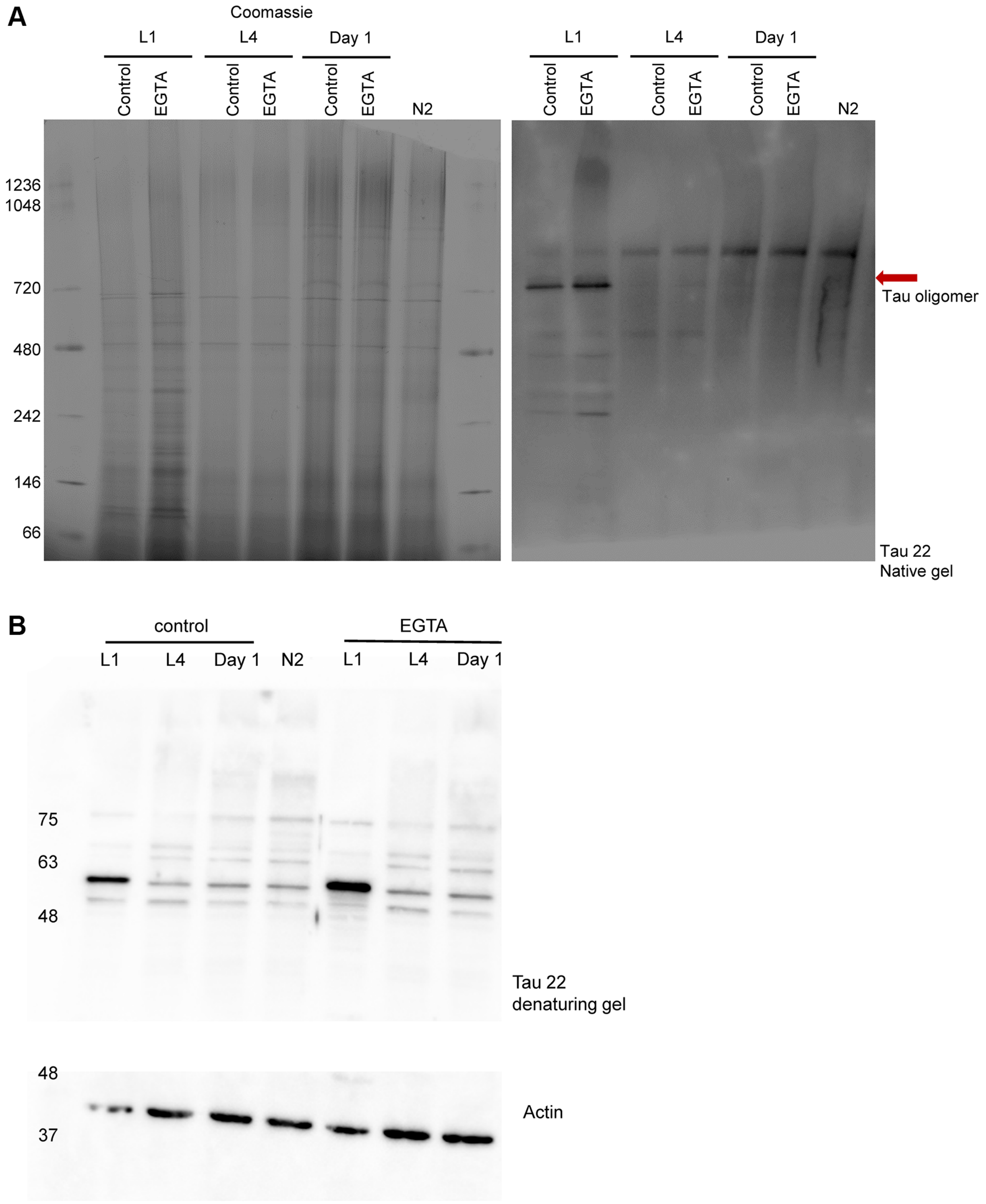

Supplementary Figure 5. EGTA supplementation does not affect the formation of tau oligomers or aggregates in *C. elegans* neurons. Worm extracts prepared from L1, L4 and 1-day transgenic tau^{wt}-expressing nematodes with or without 10 mM EGTA treatment, were resolved by (**A**) native PAGE and (**B**) SDS-PAGE and immunoblotted with Anti-Tau (T22), oligomeric antibody. Irrespective of the EGTA treatment, tau oligomers are enriched in L1 larval stage worm lysates. Band (~720 KDa marked by red arrow) on native PAGE, and (~60 KDa) on SDS-PAGE correspond to oligomeric tau species.

	Developmental stage			
	L1	L4	Day 3	Day 6
tau aggregation	No	No	Moderate	Yes
Motility defects	Yes	Yes	Yes	Yes
Mitochondrial density	low	low	high	high
Mitochondrial morphology	Smaller & globular	Smaller & globular	Smaller & globular	Smaller & globular
Mitochondrial membrane potential	Decreased	Decreased	Decreased	Decreased

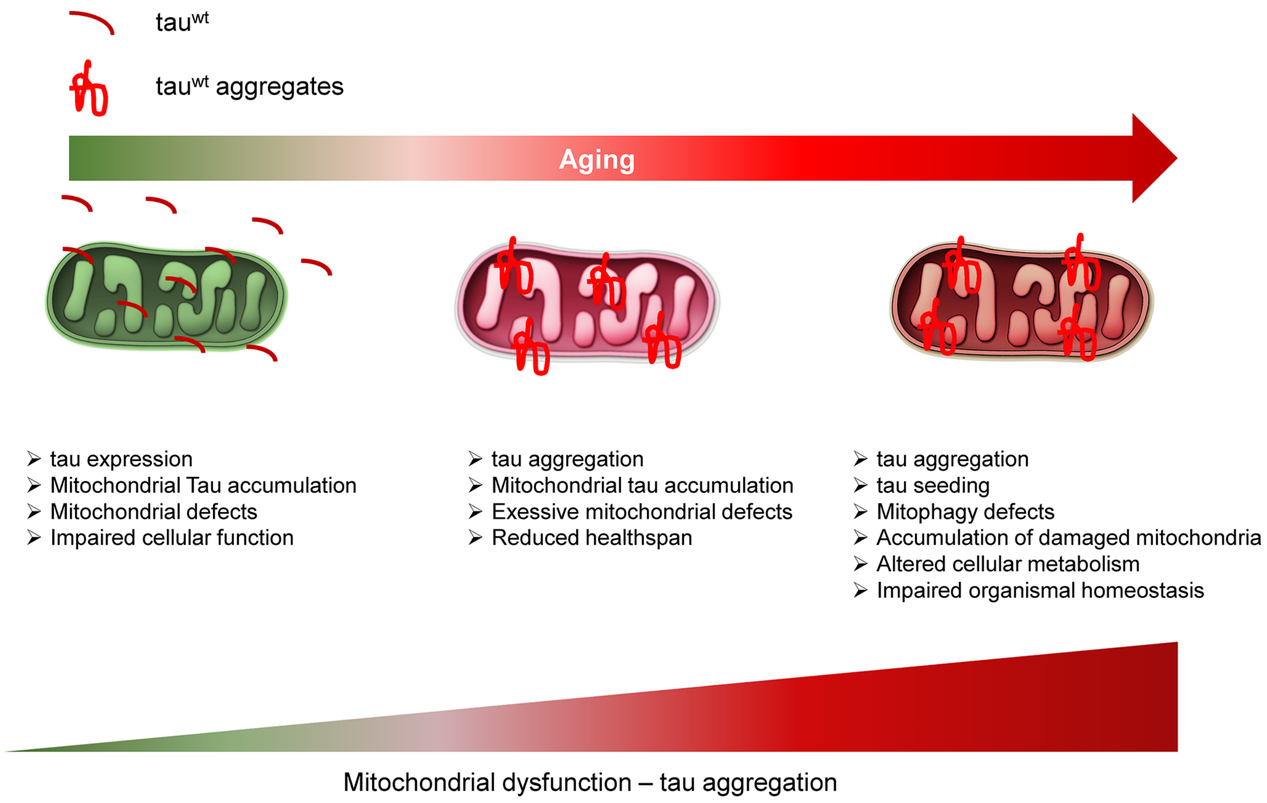

Supplementary Figure 6. Mitochondrial dysfunction is an early pathogenic feature of tauopathy. Although transgenic animals expressing tau^{wt} in neurons display excessive mitochondrial damage, which is characterized by decreased mitochondrial number, fragmented mitochondrial network and reduced membrane potential, and abnormal locomotion from L1 and L4 larval stages, tau aggregates are accumulated during adulthood. Thus, perturbed mitochondrial morphology and function manifest an early pathogenic event in the development and progression of tauophathies.
